# Reconciling evidence of oxidative weathering and atmospheric anoxia on Archean Earth

**DOI:** 10.1126/sciadv.abj0108

**Published:** 2021-09-29

**Authors:** Aleisha C. Johnson, Chadlin M. Ostrander, Stephen J. Romaniello, Christopher T. Reinhard, Allison T. Greaney, Timothy W. Lyons, Ariel D. Anbar

**Affiliations:** 1 School of Earth and Space Exploration, Arizona State University, Tempe, AZ, USA.; 2 Department of Geophysical Sciences, University of Chicago, Chicago, IL, USA.; 3 Department of Marine Chemistry and Geochemistry, Woods Hole Oceanographic Institution, Woods Hole, MA, USA.; 4 Department of Earth and Planetary Sciences, University of Tennessee—Knoxville, Knoxville, TN, USA.; 5 School of Earth and Atmospheric Sciences, Georgia Institute of Technology, Atlanta, GA, USA.; 6 Oak Ridge National Laboratory, Knoxville, TN, USA.; 7 Department of Earth and Planetary Sciences, University of California, Riverside, Riverside, CA, USA.; 8 School of Molecular Sciences, Arizona State University, Tempe, AZ, USA.

## Abstract

Evidence continues to emerge for the production and low-level accumulation of molecular oxygen (O_2_) at Earth’s surface before the Great Oxidation Event. Quantifying this early O_2_ has proven difficult. Here, we use the distribution and isotopic composition of molybdenum in the ancient sedimentary record to quantify Archean Mo cycling, which allows us to calculate lower limits for atmospheric O_2_ partial pressures (*P*O_2_) and O_2_ production fluxes during the Archean. We consider two end-member scenarios. First, if O_2_ was evenly distributed throughout the atmosphere, then *P*O_2_ > 10^–6.9^ present atmospheric level was required for large periods of time during the Archean eon. Alternatively, if O_2_ accumulation was instead spatially restricted (e.g., occurring only near the sites of O_2_ production), then O_2_ production fluxes >0.01 Tmol O_2_/year were required. Archean O_2_ levels were vanishingly low according to our calculations but substantially above those predicted for an abiotic Earth system.

## Teaser

New modeling of early Mo cycle places constraints on Archean O_2_.

## INTRODUCTION

The evolution of Earth’s atmosphere and biosphere was profoundly affected by the rise of molecular oxygen (O_2_) at the end of the Archean eon [~2.4 billion years (Ga) ago ]. Quantification of this initial rise of O_2_ remains elusive (*1*, *2*). Most constraints on the partial pressure of O_2_ (*P*O_2_) in the Archean atmosphere are upper limits, such as those derived from mass-independent fractionation of sulfur isotopes (MIF-S) [*P*O_2_ < 10^−6^ present atmospheric level (PAL); (*3*–*5*)], detrital grains of pyrite and uraninite [*P*O_2_ < 10^–3.8^ PAL; (*6*–*9*)], and Fe mobility in paleosols [*P*O_2_ < 10^–4.7^ PAL; (*10*–*13*)]. These constraints are highly informative with regard to the timing and tempo of the Great Oxidation Event (GOE), which has been dated at ~2.5 to 2.2 Ga based on when the constraints set by MIF-S and other proxies were exceeded (*14*–*18*). However, these constraints are less informative about how much O_2_, if any, was produced before the GOE, leading many to conclude that there was no O_2_ production at all [i.e., (*19*)].

In contrast, over the past 20 years, several lines of evidence have been interpreted to reflect low levels of oxidative weathering before the GOE, potentially due to transient small amounts of O_2_ [also known as “whiffs” of O_2_ or Archean oxidation events (AOEs); (*2*, *20*, *21*)]. This evidence includes concentration enrichments and shifts in the isotopic compositions of some redox-sensitive elements in marine shales (e.g., Cr, Mo, Re, Tl, and U) [(*2*) and references therein]. It is important to emphasize that these lines of evidence signify only surface redox cycling of certain elements and not necessarily oxidation by O_2_. However, the combination of multiple lines of evidence, including mass-dependent shifts in redox-sensitive light stable isotopes [e.g., (*22*–*24*)], observations from stromatolites [e.g., (*25*)], and the high organic carbon content of Archean shales [e.g., (*1*)], makes the case for early O_2_ compelling. Nevertheless, questions remain about the levels of *P*O_2_ needed to account for observed signatures of oxidative weathering and whether they can be reconciled with existing upper limits on Archean *P*O_2_.

Molybdenum (Mo) is a particularly useful proxy to address this question because its geochemical cycling and ocean isotope budget are strongly redox-dependent [summarized in (*26*)]. In the modern environment, Mo is delivered to the ocean as the soluble molybdate oxyanion (MoO_4_^2−^) following the oxidative weathering of crustal sulfides by O_2_ (*27*), with some Mo retained in weathering-resistant detrital phases (*28*). This MoO_4_^2−^ is then removed from the ocean primarily by adsorption to ferromanganese oxide minerals or by removal into highly reducing sedimentary environments rich in hydrogen sulfide (*29*–*31*). Because the marine Mo cycle relies on the supply of Mo from O_2_-induced weathering of Mo from the crust, as well as the removal of Mo into oxidizing and reducing sediments, sedimentary Mo enrichments and isotope values are powerful tracers of Earth surface redox history [e.g., (*32*, *33*)]. More to this point, Mo enrichments and isotope trends found in multiple sets of late-Archean sedimentary rocks are interpreted as indicating accumulation of low levels of O_2_ in Archean weathering and marine environments (*34*–*38*).

To explore whether oxidative weathering signatures can be reconciled with observations of an anoxic Archean atmosphere, we used observed Mo enrichments and isotope values in Archean shales to constrain a model of Mo mass balance during weathering and ocean accumulation. Using this approach, we can calculate the minimal Mo flux that needs to be delivered to the oceans. This flux, in turn, can be used to explore the viability of different oxidative weathering scenarios. Here, we explore two end-member scenarios, one in which O_2_ was well mixed in the Archean atmosphere, and another in which O_2_ was produced and accumulated strictly within localized terrestrial microbial communities in the Archean, with little to no atmospheric mixing. These models are used to establish lower limits on the amount of O_2_ present in Archean surface environments.

## RESULTS

To calculate constraints on Archean O_2_ dynamics, we explore two end-member scenarios in our calculations. First, for the purposes of organizing our thinking on Archean oxidative weathering by O_2_, we assume that O_2_ is well mixed in the atmosphere even at very low partial pressures. This assumption allows us to treat *P*O_2_ as a constant boundary condition in our weathering model, allowing us to derive lower limits on the average or equivalent *P*O_2_ responsible for stimulating sulfide weathering and mobilizing Mo. These lower limits on *P*O_2_ can then be compared to existing upper limits to test the viability of our first scenario: that oxidative weathering signatures record changes in global *P*O_2_.

Perhaps more likely, O_2_ was a trace gas during the Archean that was not well distributed in the atmosphere and, instead, existed as short-lived gas plumes and O_2_ oases in soils and shallow seas [e.g., (*39*, *40*)]. Potentially, O_2_ was so short-lived that *P*O_2_ was negligible beyond the immediate environment where O_2_ was produced. To place constraints on the O_2_ fluxes produced in these settings, we use the stoichiometry of pyrite oxidation by O_2_ to calculate the minimum O_2_ flux (Tmol/year) responsible for the Mo input recorded by sedimentary rocks. The minimum O_2_ flux in itself is a lower limit on terrestrial O_2_ production that can be compared with previous estimates of O_2_ production by microbial communities and abiotic sources to explore the viability of such a scenario as the source of oxidative weathering signatures. Below, we examine both end-members.

### End-member scenario 1: O_2_ as a globally distributed trace gas

Our approach used a Monte Carlo analysis to explore the range of plausible Mo mass balance parameters consistent with the Archean and Paleoproterozoic shale record (see Materials and Methods). Rather than reconstructing Mo mass balance from the shale record, which has many nonunique solutions, this approach explores all plausible Mo mass balance solutions and selects those that match the shale record. Paired together, Mo isotopic and concentration data are leveraged to place constraints on the total input of Mo required to sustain steady state under different marine redox scenarios. We subdivide the shale record from 3.2 to 2.0 Ga into four periods based on observed Mo concentrations and isotope values: the Paleo/Mesoarchean (3.2 to 2.8 Ga), the Neoarchean (2.8 to >2.5 Ga), the “Whiff of O_2_” sediments at 2.5 Ga (*20*), and the Paleoproterozoic (2.5 to 2.0 Ga).

**Fig. 1.**
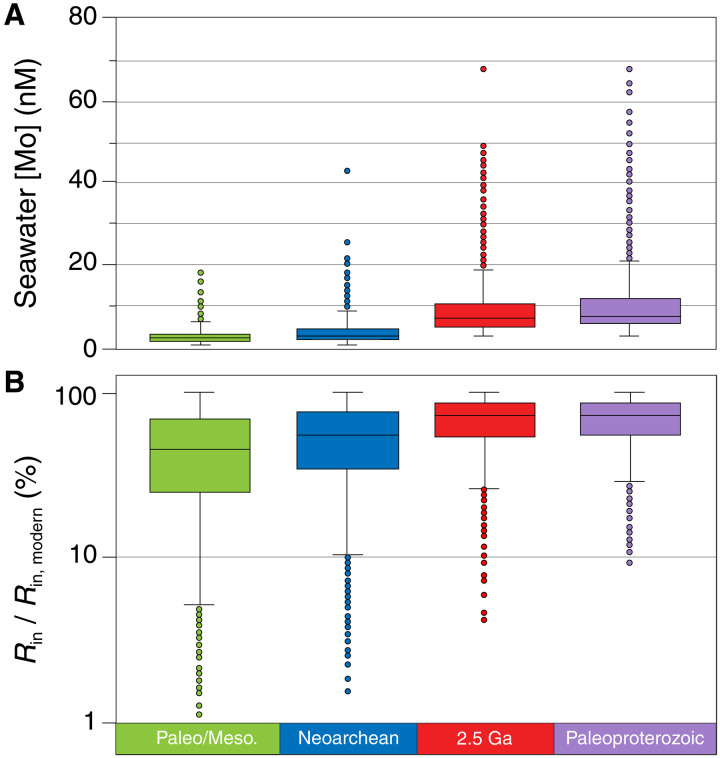
Requisite seawater [Mo] and Mo input to satisfy mass balance as indicated by ancient shales from 3.2 to 2.0 Ga. (**A**) Distribution of the seawater Mo concentrations of possible mass balance solutions, where concentrations are reported in (M × 10^9^) (**B**) Distribution of possible mass balance solutions where Mo input (*R*_in_) is plotted as a percentage of the modern riverine Mo flux ([(mol/year)_MODEL_]/[(mol/year)_MODERN_] × 100). Boxes represent the central quartiles of the mass balance solutions (Q2 and Q3) and include a horizontal line to indicate the median. Whiskers represent mass balance solutions that occur within 1.5 quartiles of the boxes; solutions that fall outside this range are plotted as points. Notably, the minimum Mo required of each solution set increases through time.

In Fig. 1, we plot the results of the mass balance model: Panel A shows the seawater concentrations of Mo for all plausible mass balance solutions. Panel B shows Mo input to the ocean (*R*_in_) from all plausible mass balance solutions, relative to the modern riverine Mo flux. All mass balance solutions from each time period require greater than 1% of the modern Mo flux, meaning they require Mo in excess of potential hydrothermal contributions (see Materials and Methods). By extension of this logic, our model indicates that our estimated hydrothermal contribution would result in Mo enrichments <2 parts per million (ppm), similar to detrital contributions. In both panels of Fig. 1, a distinct trend can be seen: The lower threshold of each mass balance solution set increases approaching and across the Archean-Proterozoic boundary. More specifically, in panel B, the minimum Mo input required to satisfy mass balance increases from ~1% of the modern Mo flux in the Archean to several percent by the end of the Neoarchean and to greater than ~10% in the Paleoproterozoic.

**Fig. 2.**
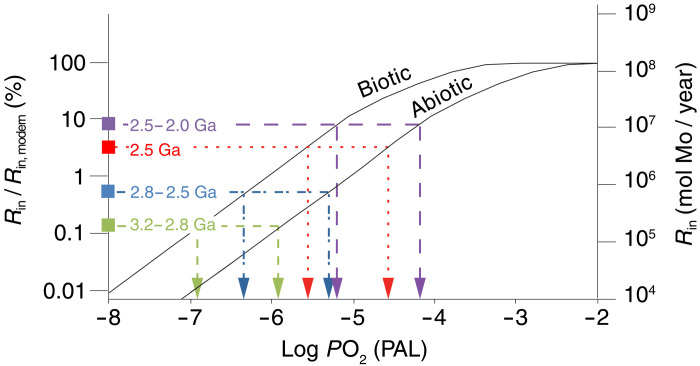
Calculation of *P*O_2_ requirements. The river flux of Mo that is delivered to the ocean over time is related to *P*O_2_ by calculating *R*_in_ (mol Mo/year) as the product of sulfide dissolution in global weathering soil profiles (see text and Materials and Methods for description of the weathering model). Modeled trends are included for abiotic sulfide oxidation by O_2_ and biotic sulfide oxidation in the presence of O_2_, with the latter considered to be roughly one order of magnitude faster based on biotic sulfide oxidation experiments (*43*). The minimum Mo requirements of all mass balance solutions for each time period are plotted on these trends, which indicate on the *x* axis the *P*O_2_ necessary to stimulate oxidative weathering and deliver the necessary flux of Mo to the ocean from crustal sulfides. Because the *R*_in_ values are minimum requirements for the mass balance solutions, the indicated *P*O_2_ values are similarly minimum estimates.

To calculate lower limits on *P*O_2_, we convert the minimum Mo input (*R*_in_) of each time period to *P*O_2_ using a previously published weathering model (Fig. 2). The model used here is that of Daines *et al.* (*41*), which calculates global sulfate production from oxidative weathering as a function of the rates of continental uplift and erosion and *P*O_2_. Specifically, their study leveraged an existing one-dimensional reaction-transport model and uplift rates from modern river sediment budgets (*41*). We updated the sulfide oxidation kinetics of the model using the rate law of Johnson *et al.* (*42*), which specifically determined reaction kinetics at levels of *P*O_2_ relevant to this study (<10^−5^ PAL *P*O_2_). We then multiplied the global sulfate input by the Mo/S ratio of modern rivers (*27*) as a proxy for the Mo/S ratio of crustal sulfides to calculate the *P*O_2_-dependent global Mo flux from oxidative weathering of sulfides by O_2_ (Fig. 2).

In the modern environment, biology is well known to accelerate mineral sulfide oxidation and is thus important to consider in these weathering calculations. However, because the influence of biology on the weathering rate is not explicitly considered in the model, we include an estimate for biologically mediated sulfide oxidation, which some experiments have identified as being ~1 order of magnitude faster than abiotic sulfide oxidation by O_2_ [e.g., (*43*)].

In Fig. 2, we obtain lower limits on *P*O_2_ by comparing the derived Mo input fluxes as a function of *P*O_2_ to our minimum Mo input estimates from each time period, following a correction for potential hydrothermal contribution (see Materials and Methods). From the Paleoarchean through the Mesoarchean (3.2 to 2.8 Ga), Mo enrichments correspond to a lower limit on *P*O_2_ of ~10^–6.9^ PAL. Neoarchean enrichments (2.8 to 2.55 Ga) correspond to a lower limit of *P*O_2_ ≥ 10^–6.3^ PAL. The enrichments at 2.5 Ga deviate even more substantially from crustal values, requiring *P*O_2_ ≥ 10^–5.6^ PAL. Paleoproterozoic enrichments (2.4 to 2.0 Ga) are the largest and contain the most isotopically heavy values, requiring *P*O_2_ ≥ 10^–5.2^ PAL.

**Fig. 3.**
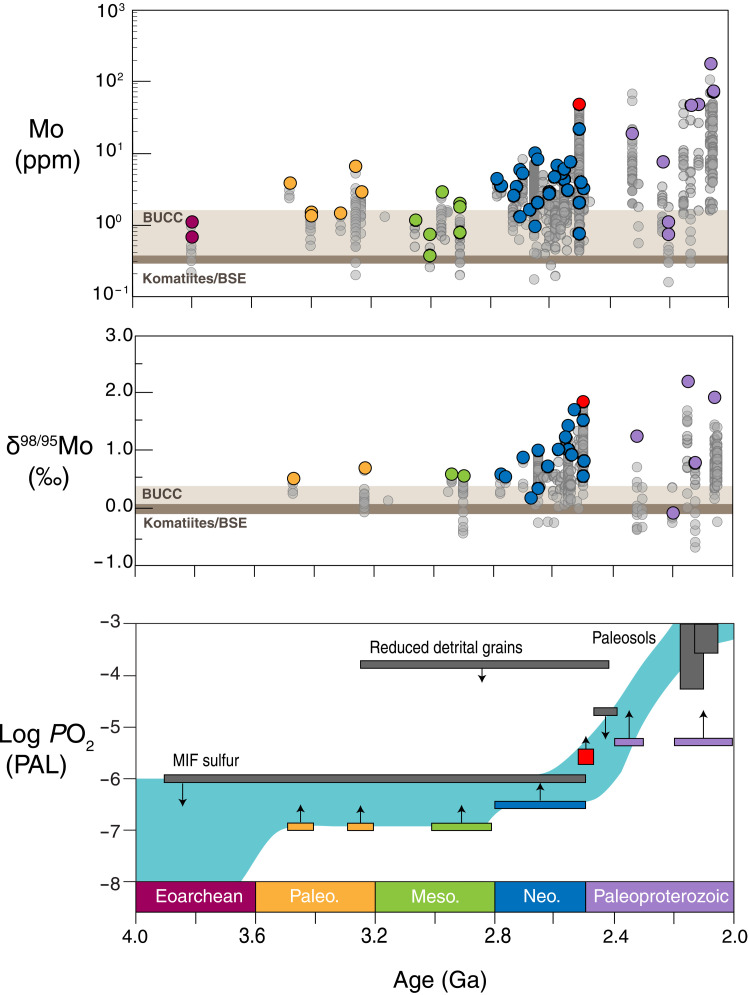
Archean and Paleoproterozoic estimates of equivalent atmospheric *P*O_2_ derived from Mo concentration enrichments and isotope values in black shales. (**A** and **B**) Compiled data for Mo (ppm) and δ^98/95^Mo (‰) in Archean- and Paleoproterozoic-age black shales compared to bulk upper continental crust (BUCC) and values for komatiites and bulk silicate Earth (BSE) (*68*–*70*). The maximum value for each particular shale unit is colored, while all other data from each unit are gray (*20*, *32*, *34*, *35*, *63*, *71*–*84*). (**C**) *P*O_2_ requirements of mass balance solutions that are consistent with Mo concentration enrichments and isotope values in the geologic record, where *P*O_2_ is in units of PAL and compared to previously published *P*O_2_ constraints [5 (<10^−6^ PAL); 6 (<10^–3.8^ PAL before 2.415 Ga); 10 (<10^–4.7^ PAL at ~2.46 Ga, 10^–4.3^ to 10^–1.8^ PAL at ~2.15 Ga, 10^–3.6^ to 10^–1.0^ PAL at ~2.08 Ga)].

In Fig. 3, we use the compiled Mo enrichment and isotope values from the shale record to determine over which periods of time we are able to directly place lower limits on *P*O_2_. We subdivide each era of Archean time into 100-million-year time bins and place lower limits on *P*O_2_ in each interval for which both concentration enrichments and isotope data exist above crustal values. The resulting *P*O_2_ constraints through time thus reflect the temporal spread of available data obtained from the geologic record, which generally decreases further back through time as there are fewer suitable samples. Because Paleo and Mesoarchean Mo enrichments are so close to crustal values, we cannot rule out that these earliest Mo “enrichments” were deposited by an anomalous mechanism (i.e., increased Mo sourcing from hydrothermal activity) or that the composition of the upper crust was different at that time relative to our current estimates, leading to false positives for O_2_. However, with our best current assumptions, Mo supply from low-level oxidative weathering remains the most parsimonious explanation for the observed shale enrichments.

The lowest *P*O_2_ estimates presented here are those that consider biologically mediated pyrite oxidation (*P*O_2_ ≥ 10^–6.9^ PAL; Figs. 2 and 3C). These estimates are consistent with previously published upper limits on *P*O_2_, which are ~10 times higher (Figs. 2 and 3C). It is thus plausible that the oxidation of crustal sulfide grains was a biologically mediated process for much, if not all, of the Archean [see also (*44*)]. However, because we also find that the *P*O_2_ required in abiotic scenarios is near existing upper limits, biologically mediated sulfide oxidation may not have been required.

These lower limits for *P*O_2_ agree well with *P*O_2_ upper limits derived from MIF-S, detrital grains, and paleosols (Fig. 3 and Table 1). Together, these constraints could define a plausible Archean O_2_ curve that can account for the seemingly paradoxical presence of oxidative weathering signatures produced under a reducing atmosphere. Similar to the Mo record, these calculated lower limits also increase throughout the Archean, which may imply that O_2_ production increased or O_2_ sinks decreased during the time leading up to the GOE. These implications are discussed further in later sections.

**Table 1.** Summary of estimates of O_2_ levels in the Archean atmosphere.

**Archean O_2_** **estimate**	**Global or** **local**	**Proxy**	**Minimum or** **maximum** **estimate**	**Author**
*P*O_2_ < 10^−6^	Global	MIF-S anomalies	Maximum	(*3*–*5*)
*P*O_2_ < 10^–3.8^ PAL	Global	Detrital pyrite and uraninite	Maximum	(*6*–*9*)
*P*O_2_ < 10^–4.7^ PAL	Global	Fe mobility in paleosols	Maximum	(*10*–*13*)
*P*O_2_ > 10^–6.9^ PAL	Global	Mo in shales	Minimum	This study
>0.01 Tmol O_2_/year	Local	Mo in shales	Minimum	This study

It may seem counterintuitive that oxidative weathering of sulfides was active in the Archean, given the presence of detrital pyrite grains in many Archean sedimentary successions [e.g., (*6*, *45*, *46*)]. As shown in Fig. 2, modeled sulfide oxidation can be subdivided into two regimes: O_2_-limited at low *P*O_2_ and sulfide-limited at high *P*O_2_. The transition between these two regimes marks a shift in the limiting reactant (O_2_ versus pyrite availability) for global sulfide oxidation. In modern highly oxygenated environments, rates of sulfide oxidation are limited by the supply of pyrite and other sulfides in weathering soils, resulting in a zero-order dependence on *P*O_2_ (*47*). At lower *P*O_2_, the rate of sulfide oxidation slows to the point where the net rate is limited by O_2_ diffusion into weathering soils. Under these latter conditions, the supply of sulfide minerals is not limiting so that detrital pyrite can survive weathering in soils by not fully oxidizing. In the modeling presented here, the transition between these regimes occurs at around 10^−4^ to 10^−3^ PAL, in broad agreement with previous calculations for the oxidation of detrital grains [*P*O_2_ < 10^–3.8^ PAL; (*6*–*9*)] and global sulfide weathering models (*41*, *47*). Notably, the time periods examined in this study all fall within the O_2_-limited sulfide oxidation regime.

Consistent with this finding, diamictites from 2.90 to 2.43 Ga show only small degrees of Mo mobilization (*48*, *49*). Retention of Mo in diamictites is also expected because Mo^4+^ often substitutes for Ti^4+^ in titaniferous, weathering-resistant minerals, meaning only a fraction of crustal Mo is hosted in sulfides and mobilized during oxidative weathering (*28*, *50*). For instance, Greaney *et al.* (*28*) estimated that ~60% of the Mo hosted in the modern upper continental crust could be contained within sulfide minerals. If 1% of sulfide-hosted Mo was liberated due to oxidative weathering, the regolith sampled by diamictites would only record a 0.6% change in total Mo content or less if the diamictites also sampled primary bedrock. In short, the modeling presented here demonstrates that the Mo required to satisfy our mass balance model can be supplied at each stage of the Archean without fully dissolving sulfide minerals, which is consistent with other evidence typically interpreted to support a low-O_2_ atmosphere.

### End-member scenario 2: O_2_ as a nonglobally distributed trace gas

As mentioned previously, it is unlikely that O_2_ was well distributed in the atmosphere at levels approaching ~10^−7^ PAL *P*O_2_. To sustain these levels, primary production would have to match or exceed the modern O_2_ flux to balance the rapid supply of reduced gases [e.g., (*4*, *51*)]. It is also commonly thought that ~10^−7^ PAL *P*O_2_ is a point of atmospheric instability, either resulting in a return to a more reduced state or the oxygenation of the atmosphere [i.e., (*52*)]. It is much more likely that O_2_ was produced locally in soils and shallow seas by microbial communities and rapidly consumed locally by reduced materials before entering the greater atmosphere [e.g., (*39*, *53*, *54*)]. In such a scenario, our equivalent *P*O_2_ estimates are still valid as lower limits for the *P*O_2_ experienced by some soils but likely do not reflect the atmosphere in the way that *P*O_2_ inferred from MIF-S does.

To evaluate the viability of local O_2_ production as the source of oxidative weathering signatures, we can calculate the flux of O_2_ implied by Mo and S delivery to the ocean and compare to previous estimates of terrestrial primary productivity. As shown in Fig. 1, all mass balance solutions require some amount of minimum Mo input to the ocean, measured in mol Mo/year. In the previous section, we relate the minimum Mo input from rivers to sulfate input from sulfide weathering using the Mo/S ratio of modern rivers (*27*). Using the minimum sulfate input for each time period, we can then use the stoichiometry of pyrite oxidation by O_2_ to calculate how many moles of O_2_ were consumed by sulfide oxidation, assuming that all oxidizing power was converted to SO_4_^2−^ and not Fe^3+^

$${\text{FeS}}_{2}+7/2\;O_{2}+H_{2}O\to {\text{Fe}}^{2+}+2{{\text{SO}}_{4}}^{2‐}+2H^{+}$$ (1)

This approach indicates that the rate of O_2_ consumed by terrestrial sulfide oxidation was on the order of ≥0.02 Tmol O_2_/year from 3.5 to 2.8 Ga, increasing to ≥0.06 Tmol O_2_/year from 2.8 to 2.55 Ga. At 2.5 Ga, the sudden shift in marine Mo enrichments corresponds to an O_2_ flux of ≥0.4 Tmol O_2_/year. Paleoproterozoic enrichments require ≥1.0 Tmol O_2_/year consumed by terrestrial sulfide oxidation. These estimates are well within the range of proposed O_2_ production rates by terrestrial benthic communities, which extend up to 10 Tmol O_2_/year even at 1% or less of modern crustal coverage (*53*), and are far below modern net primary productivity (~10 Tmol O_2_/year) (*55*). Therefore, local O_2_ production by terrestrial microbial communities is a viable mechanism for generating Archean oxidative weathering signatures.

## DISCUSSION

### A framework for Archean oxidative weathering

**Fig. 4.**
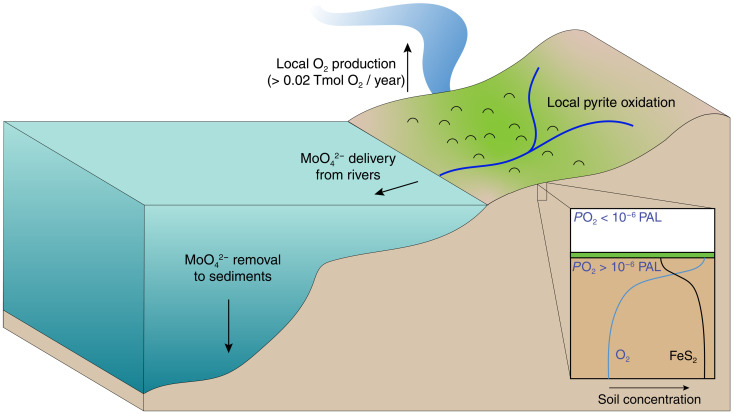
An emergent view of Archean terrestrial O_2_ production. It is likely that oxidative weathering signatures such as Mo enrichments in shales preceded atmospheric oxygenation due to local O_2_ production and consumption in terrestrial environments. Shallow soils in proximity to microbial communities (green in the figure) may have experienced greater than ~10^−7^ PAL *P*O_2_, which was capable of partially oxidizing sulfide grains and delivering trace metals such as Mo to rivers and marine environments in excess of hydrothermal or detrital contributions. Until the late Archean, these O_2_ fluxes had very little effect on atmospheric redox, which remained <10^−6^ PAL *P*O_2_ in the presence of rapidly supplied reductants (O_2_ sinks).

Our study is an example of how new estimates of *P*O_2_ and O_2_ production can be derived from existing geochemical data, reconciling observations of Archean oxidative weathering with evidence of a reducing atmosphere. We imagine that O_2_ production by terrestrial microbial communities stimulated highly localized oxidative weathering, resulting in the delivery of redox-sensitive trace metals to marine environments (Fig. 4). For most of the Archean, these O_2_ sources were greatly exceeded by O_2_ sinks and were fairly unimportant in determining atmospheric redox (*5*, *51*). However, the Neoarchean observed a substantial rise in O_2_ consumption by oxidative weathering of sulfides, primarily surrounding the AOE at 2.5 Ga (*20*, *21*, *44*). This pulse of oxidative weathering could have been caused by increased landmass [e.g., (*56*)], increased O_2_ production, or both in the instance where increased landmass promoted terrestrial colonization and the exposure of crustal sulfides to weathering. One could also imagine that if AOEs were tied to solid Earth drivers such as landmass, then those same mechanisms may have played a role in triggering the GOE [e.g., (*55*)]. Future studies leveraging geochemical data to learn about ancient O_2_ are well positioned to verify these mechanisms.

At the same time that O_2_ was rising in the environment and stimulating sulfide weathering, Mo began accumulating in the oceans at biologically relevant levels. Most of the mass balance solutions from our model indicate that for most of the Archean, seawater concentrations of Mo were below 5 nM (Fig. 1A). This threshold is notable because it was previously identified by culturing experiments to be the limit below which Mo becomes limiting for N_2_ fixation in cyanobacteria (*57*). If Mo was favored as an enzymatic cofactor by this time by N_2_ fixers [e.g., (*58*)], our results imply that periods of biological Mo limitation on N fixation (seawater [Mo] < 5 nM) may have occurred frequently during Archean time, at least on a regional scale (*59*). Following 2.5 Ga, most mass balance solutions indicate that seawater concentrations of Mo were greater than 5 nM, perhaps ushering in a period where Mo was less likely to be limiting for N_2_ fixation in marine environments and representing a potentially important change to the global N cycle. To fully explore this possibility, however, better understanding of large-scale interconnections among Mo availability, biological N fixation and associated thresholds of Mo limitation, contributions from alternative nitrogenase pathways, and the importance of phosphorus (P) limitation is needed. Nevertheless, this feedback may have been important for marine productivity as the rise of O_2_ in the marine environment would have promoted loss of fixed N to the atmosphere as N_2_ (*24*, *57*, *60*).

Last, our study demonstrates that for much of the Archean, the frequent use of “anoxic” should not imply a complete absence of O_2_. Low levels of O_2_ may have persisted below the MIF-S threshold for hundreds of millions of years before the GOE, with important environmental and biological consequences. More generally, the O_2_ lower limits derived here are a first step toward a more nuanced understanding of Archean levels of O_2_.

## MATERIALS AND METHODS

### Monte Carlo analysis

To find viable mass balance solutions to Archean Mo cycling, we use a Monte Carlo model to generate hypothetical data, given a series of source and sink constraints, and use Mo concentration and isotope values from ancient shales to “filter” out implausible results. Our model balances Mo input from rivers and hydrothermal sources against Mo burial in three sedimentary sinks [modified from (*26*)]: (i) oxide-bearing sediments (Fe oxides, Mn oxides), which are typically deposited beneath oxygenated bottom waters [>10 μM (O_2_)]; (ii) sulfidic at depth (SAD) sediments, which are typically deposited beneath suboxic bottom waters and begin to accumulate H_2_S in the sediment porewater [<10 μM (O_2_), (H_2_S) <11 μM]; and (iii) euxinic sediments, which are deposited beneath sulfidic bottom waters [<10 μM (O_2_), (H_2_S) >11 μM].

We consider a steady-state mass balance model of Mo concentrations in seawater as a function of Mo inputs and outputs [modified from (*61*)]. The key governing equations are

$$R_{\text{out},i}=b_{i}\times {\left[\text{Mo}\right]}_{\text{sw}}\times A_{i}$$ (2)

$$R_{\text{in}}=R_{\text{out}}=\sum R_{\text{out},i}$$ (3)

$$f_{i}=R_{\text{out},i}/R_{\text{out}}$$ (4)

$$\delta^{98}{\text{Mo}}_{\text{in}}=\delta^{98}{\text{Mo}}_{\text{out}}=\sum (f_{i}\times \delta^{98}{\text{Mo}}_{i})$$ (5)

where *R*_out,i_ is the rate of Mo removal (mol year^−1^) to each sedimentary environment i (i.e., oxide-bearing, SAD, or euxinic); *b*_i_ is an effective rate constant for removal of Mo (liter km^−2^ year^−1^); [Mo]_sw_ is the concentration of Mo in seawater (M); *A*_i_ is the depositional area of that sedimentary environment (km^2^) (where ∑*A*_i_ = total areal extent of the modern seafloor); *R*_in_ and *R*_out_ are the total input and output rates of Mo to and from the oceans, respectively (mol year^−1^); *f*_i_ is the fraction of the total Mo output represented by each particular sedimentary environment; and δ^98^Mo (‰) are the isotope compositions of each sedimentary environment (where δ^98^Mo = (^98/95^Mo_sample_/^98/95^Mo_standard_ − 1) × 1000).

The isotope compositions are additionally constrained by the relationships δ^98^Mo_euxinic_ = δ^98^Mo_sw_, δ^98^Mo_oxide_ = δ^98^Mo_sw_ + ∆_sw-oxide_, and δ^98^Mo_SAD_ = δ^98^Mo_sw_ + ∆_sw-SAD_. Here, ∆_sw-i_ are respective isotope fractionation factors inferred from field observations and laboratory experiments (Table 2). We assume that δ^98^Mo_euxinic_ = δ^98^Mo_sw_ because it is likely that highly reducing sediments at this time could quantitatively remove the small amounts of Mo from overlying seawater. For ∆_sw-oxide_, we can subdivide into two sediment types with distinct fractionation behavior: Mn oxide–bearing sediments (∆_sw-Mn_) and Fe oxide–bearing sediments (∆_sw-Fe_). This step is additionally important to account for the possibility of sediments deposited beneath ferruginous [Fe(II)-rich] water columns, which may preserve Fe oxides in the absence of O_2_ and H_2_S. For Fe oxide–bearing sediments, we select a fractionation factor of 1.25‰, a median value between the fractionation factors of magnetite (Δ^98^Mo = 0.83 ± 0.60‰), ferrihydrite (ferrihydrite (Δ^98^Mo = 1.11 ± 0.15‰), goethite (Δ^98^Mo = 1.40 ± 0.48‰), and hematite (Δ^98^Mo = 2.19 ± 0.54‰) (*62*).

**Table 2.** Parameters used in Monte Carlo analysis.

**Parameter**	**Range**	**References**
Depositional area (*A*_Di_) (% total)		
Fe oxides	0.01–100	
Mn oxides	0.01–10	(*38*)
SAD	0.01–100	
Euxinic	0.01–5	(*61*)
Burial rate constant (*b*_i_) (liter km^−2^ year^−1^)		
Fe oxides	1.0–3.0 (× 10^6^)	(*32*)
Mn oxides	1.0–3.0 (× 10^6^)	(*32*)
SAD	1.75–4.25 (× 10^8^)	(*32*)
Euxinic	0.6–1.8 (× 10^9^)	(*32*)
Fractionation factor (Δ_SW-i_) (‰)		
Fe oxides	1.25	(*62*)
Mn oxides	3	(*85*)
SAD	0.7	(*26*)
Euxinic	0	(*26*)
Seawater Mo ([Mo]_SW_) (M)	0.01–105 (× 10^−9^)	
River δ^98/95^Mo (δ^98/95^Mo_in_) (‰_in_)	0–0.7	(*26*)

The *b*_i_ values are calibrated from modern settings and are derived as follows: *R*_out,i_ = *r*_i_ × [Mo]_i_ × *A*_i_, where *r*_i_ is the Mo burial rate for sediment i (kg km^−2^ year^−1^), and [Mo]_i_ = *a*_i_ × [Mo]_sw_, where *a*_i_ is a distribution coefficient relating sediment and seawater concentrations (liter kg^−1^). Hence, *R*_out,i_ = *r*_i_ × *a*_i_ × [Mo]_sw_ × *A*_i_ = *b*_i_ × [Mo]_sw_ × *A*_i_, where *b*_i_ = *r*_i_ × *a*_i_.

Using this framework, [Mo]_sw_ can be calculated from derived parameters when combined with key measurements such as δ^98^Mo_euxinic_ in ancient black shales. However, the derived parameters, particularly *A*_i_ and *b*_i_, encompass considerable uncertainties, and the system of equations is underconstrained so that there are many nonunique solutions. There are also uncertainties about overall ocean mass balance because *R*_in_ is not well constrained.

To explore this parameter space, we use a Monte Carlo analysis whereby *b*_i_, [Mo]_sw_, δ^98^Mo_in_, and *A*_i_ are varied, assuming either a uniform probability distribution or a normal probability distribution across all plausible ranges (Table 2). For instance, [Mo]_sw_ is varied from extremely low concentrations (0.01 × 10^−9^ M) to modern marine concentrations (105 × 10^−9^ M) using a uniform probability distribution that reflects our lack of constraints on ancient seawater [Mo]. For compositions of sediment that had a low likelihood of extending beyond continental margins (euxinic and Mn oxide–bearing sediments), we limit *A*_D_ ranges to <5% and <10% of the total ocean floor, respectively. Because the deep ocean was likely ferruginous and may have contained SAD sediments or Fe oxide–bearing sediments, depending on S availability, we explore the full range of depositional area for these sediment compositions. Burial rate constants (*b*) were sampled using a normal distribution because they are measured values. For *b* value ranges, uncertainty was set at ~±25% of the measured modern value to represent not only analytical uncertainty but also uncertainty with respect to how these values apply to Archean marine sediments. This analysis produces several million solutions to the mass balance equations, which we then filter using constraints from the modern environment and geologic record to eliminate implausible results.

For modeling purposes, the Archean is subdivided into two categories based on geologic eras of distinct shale-hosted Mo enrichment concentrations and isotopic values: the Paleo and Mesoarchean, and the Neoarchean. The whiff of O_2_ at 2.5 Ga comprises a third category due to its anomalous nature and is not included with other Neoarchean sediments. For comparative purposes, we also include a fourth category for the Paleoproterozoic. Compiled data can be found in the Supplementary Materials.

### Filtering mass balance solutions

Filtering of mass balance solutions for each time period varied according to available constraints (Table 3). The first filter removed all scenarios that required a greater flux of Mo to the ocean than that which is observed in the modern environment (Table 3). The reasoning behind this constraint is that in the modern highly oxygenated environment, crustal sulfides dissolve completely, and thus, Mo delivery from oxidative weathering is maximized. At lower concentrations of atmospheric O_2_, we assume that crustal Mo delivery could only be less than the modern.

**Table 3.** Parameters used for filtering.

**Parameter**	**Range**	**References**
Seawater δ^98/95^Mo (‰)		See compiled data sources
Paleo-Mesoarchean	0.45–0.75	
Neoarchean	0.90–1.20	
Whiff (2.5 Ga)	1.50–1.80	
Paleoproterozoic	1.30–2.00	
Maximum shale Mo enrichment (μg g^−1^)		See compiled data sources
Paleo-Mesoarchean	2–5	
Neoarchean	5–10	
Whiff (2.5 Ga)	35–50	
Paleoproterozoic	40–70	
Maximum shale sedimentary rate (g cm^−2^ year^−1^)	0.0125	(*61*)
Minimum shale sedimentary rate (g cm^−2^ year^−1^)	0.00125	(*61*)
Modern river Mo flux (*F*_in_) (mol year^−1^)	1.35 × 10^8^	

The second filter removed all scenarios that did not produce an isotopic composition in euxinic sediments within the heaviest range typically observed for a given time period (Table 3). We assumed that ancient euxinic sediments captured δ^98/95^Mo_SW_ values at the time of deposition [although this is not always the case; see Kendall *et al.* (*26*) and references therein], which allowed the second filter to remove model solutions that produced δ^98^Mo_SW_ values outside the analytical uncertainty of the heaviest shale δ^98/95^Mo values. The reasoning here is that the Mo contained within shales is a mix of detritally hosted Mo and authigenic Mo, meaning that the δ^98/95^Mo values of these shales are always equal to or lighter than contemporaneous δ^98/95^Mo_SW_ (*63*). The heaviest values of δ^98/95^Mo in Archean euxinic shales are thus those with the least crustal influence and are the most likely to have captured ancient δ^98/95^Mo_SW_.

A third filter removed scenarios where the depositional area of euxinic sediments exceeded the depositional area of SAD sediments. This logic has been applied before to Mo mass balance solutions [i.e., (*64*)] to avoid unrealistic scenarios where hypothetical euxinic sediments coexist with oxide-bearing sediments and lack the SAD sediments that would exist in the transition between. We expect that the formation of euxinic and SAD sediments scales together; thus, in all of our final mass balance solutions, *A*_DSAD_ > *A*_DE_.

Last, a fourth filter removed all scenarios that did not reproduce the concentrations of Mo within the range observed in ancient euxinic shales (Table 3). For each condition, the flux of Mo to euxinic sediments is divided by a maximum and minimum mass accumulation rate to calculate the range of possible authigenic enrichments. If a mass balance solution produced both a minimum and maximum enrichment that were outside the range observed in shales, it was removed. All ranges in Table 3 capture the maximum enrichments observed in black shales during a given time period, including variability between similarly aged rock units. The lower cutoff is 2 μg g^−1^ because enrichments <2 may simply reflect Mo in detrital grains. The range of sedimentation rates was modified from those used by Reinhard *et al.* (*61*), which were derived from the sedimentation rates of modern anoxic/euxinic basins connected to the open ocean (e.g., Cariaco basin). Here, we vary sedimentation rate by an order of magnitude to ensure that all potentially realistic scenarios are captured by the modeling.

### Hydrothermal input

With the lower limits on Mo input to the ocean from Fig. 1, it is now possible to place constraints on oxidative weathering and, by extension, *P*O_2_. First, we subtract the potential hydrothermal Mo input to prevent overestimating Mo input from oxidative weathering. Where data have been collected in the modern environment, it appears that both high-temperature and low-temperature fluid alterations of the lithosphere can result in Mo-bearing fluids (*27*, *65*, *66*). Low-temperature fluids are a important source of Mo to the modern ocean, on the order of 13% of Mo input from rivers (*27*). However, there is evidence that suggests that the Mo in low-temperature fluids is sourced from the reductive dissolution of Mo-bearing sediments overlying oceanic crust (*67*), and thus, low-temperature fluids more closely represent a failed Mo sink rather than a Mo source over geologic time scales. High-temperature fluids were once estimated to contribute 1% of the Mo input from rivers (*27*, *65*), although they are now established to be a net sink in the modern environment (*27*). It is not yet clear whether the Mo released from high-temperature vents is inherited from seawater or from leaching of the basalt, which makes it difficult to extrapolate to Archean oceans. If Mo is readily leached from basalt during high-temperature alteration, hydrothermal activity could have been an important anoxic source of Mo to Archean seawater. Consequently, our *R*_in_ values would overestimate the role of oxidative weathering in supplying Mo to the ocean.

To account for the possibility that Archean hydrothermal sources of Mo were important, we subtract 1% of the modern Mo input from our *R*_in_ values to estimate the contribution of hydrothermal sources, based on the values of Wheat *et al.* (*66*). This approach more accurately calculates the minimum Mo input from oxidative weathering. Following this correction, we selected the lowest Mo flux for *P*O_2_ and O_2_ flux calculations.

## Supplementary Materials

### This PDF file includes:

References
