# Supplementary material for: Reconciling evidence of oxidative weathering and atmospheric anoxia on Archean Earth
Source: Sci Adv. 2021 Sep 29;7(40):eabj0108. doi: 10.1126/sciadv.abj0108 (PMC8480925; doi:10.1126/sciadv.abj0108)
Supplement: Supplementary file 1 — References [file sciadv.abj0108_sm.pdf]

Science Advances
AAAS

Supplementary Materials for

Reconciling evidence of oxidative weathering and atmospheric anoxia on Archean Earth

Aleisha C. Johnson*, Chadlin M. Ostrander, Stephen J. Romaniello, Christopher T. Reinhard, Allison T. Greaney, Timothy W. Lyons, Ariel D. Anbar

*Corresponding author. Email: acjohnson@uchicago.edu

Published 29 September 2021, *Sci. Adv.* **7**, eabj0108 (2021)
DOI: 10.1126/sciadv.abj0108

The PDF file includes:

References

Other Supplementary Material for this manuscript includes the following:

Data S1

REFERENCES AND NOTES

1. T. W. Lyons, C. T. Reinhard, N. J. Planavsky, The rise of oxygen in Earth's early ocean and atmosphere. *Nature* **506**, 307–315 (2014).
2. C. M. Ostrander, A. C. Johnson, A. D. Anbar, Earth's first redox revolution. *Annu. Rev. Earth Planet. Sci.* **49**, 337–366 (2021).
3. A. A. Pavlov, J. F. Kasting, Mass-independent fractionation of sulfur isotopes in Archean sediments: Strong evidence for an anoxic Archean atmosphere. *Astrobiology* **2**, 27–41 (2002).
4. K. Zahnle, M. Claire, D. Catling, The loss of mass-independent fractionation in sulfur due to a Palaeoproterozoic collapse of atmospheric methane. *Geobiology* **4**, 271–283 (2006).
5. D. C. Catling, K. J. Zahnle, The Archean atmosphere. *Sci. Adv.* **6**, eaax1420 (2020).
6. J. E. Johnson, A. Gerpheide, M. P. Lamb, W. W. Fischer, O2 constraints from Paleoproterozoic detrital pyrite and uraninite. *GSA Bull.* **126**, 813–830 (2014).
7. D. E. Grandstaff, Origin of uraniferous conglomerates at Elliot Lake, Canada and Witwatersrand, South Africa: Implications for oxygen in the Precambrian atmosphere. *Precambrian Res.* **13**, 1–26 (1980).
8. H. E. Frimmel, Archaean atmospheric evolution: Evidence from the Witwatersrand gold fields, South Africa. *Earth Sci. Rev.* **70**, 1–46 (2005).
9. C. T. Reinhard, R. Raiswell, C. Scott, A. D. Anbar, T. W. Lyons, A late Archean sulfidic sea stimulated by early oxidative weathering of the continents. *Science* **326**, 713–716 (2009).
10. Y. Kanzaki, T. Murakami, Estimates of atmospheric O2 in the Paleoproterozoic from paleosols. *Geochim. Cosmochim. Acta* **174**, 263–290 (2016).
11. T. Murakami, B. Sreenivas, S. D. Sharma, H. Sugimori, Quantification of atmospheric oxygen levels during the Paleoproterozoic using paleosol compositions and iron oxidation kinetics. *Geochim. Cosmochim. Acta* **75**, 3982–4004 (2011).
12. W. Yang, H. D. Holland, The Hekpoort paleosol profile in Strata 1 at Gaborone, Botswana: Soil formation during the Great Oxidation Event. *Am. J. Sci.* **303**, 187–220 (2003).
13. R. Rye, H. D. Holland, Paleosols and the evolution of atmospheric oxygen: A critical review. *Am. J. Sci.* **298**, 621–672 (1998).
14. G. Luo, S. Ono, N. J. Beukes, D. T. Wang, S. Xie, R. E. Summons, Rapid oxygenation of Earth's atmosphere 2.33 billion years ago. *Sci. Adv.* **2**, 1600134 (2016).

15. A. P. Gumsley, K. R. Chamberlain, W. Bleeker, U. Söderlund, M. O. de Kock, E. R. Larsson, A. Bekker, Timing and tempo of the Great Oxidation Event. *Proc. Natl. Acad. Sci. U.S.A.* **114**, 1811–1816 (2017).
16. P. Philippot, J. N. Ávila, B. A. Killingsworth, S. Tessalina, F. Baton, T. Caquineau, E. Muller, E. Pecoits, P. Cartigny, S. V. Lalonde, T. R. Ireland, C. Thomazo, M. J. van Kranendonk, V. Busigny, Globally asynchronous sulphur isotope signals require re-definition of the Great Oxidation Event. *Nat. Commun.* **9**, 2245 (2018).
17. M. R. Warke, T. Di Rocco, A. L. Zerkle, A. Lepland, A. R. Prave, A. P. Martin, Y. Ueno, D. J. Condon, M. W. Claire, The Great Oxidation Event preceded a Paleoproterozoic "snowball Earth". *Proc. Natl. Acad. Sci. U.S.A.* **117**, 13314–13320 (2020).
18. S. W. Poulton, A. Bekker, V. M. Cumming, A. L. Zerkle, D. E. Canfield, D. T. Johnston, A 200-million-year delay in permanent atmospheric oxygenation. *Nature* **592**, 232–236 (2021).
19. W. W. Fischer, J. Hemp, J. E. Johnson, Evolution of oxygenic photosynthesis. *Annu. Rev. Earth Planet. Sci.* **44**, 647–683 (2016).
20. A. D. Anbar, Y. Duan, T. W. Lyons, G. L. Arnold, B. Kendall, R. A. Creaser, A. J. Kaufman, G. W. Gordon, C. Scott, J. Garvin, R. Buick, A whiff of oxygen before the great oxidation event?. *Science* **317**, 1903–1906 (2007).
21. M. C. Koehler, R. Buick, M. A. Kipp, E. E. Stüeken, J. Zaloumis, Transient surface ocean oxygenation recorded in the∼ 2.66-Ga Jeerinah Formation, Australia. *Proc. Natl. Acad. Sci. U.S.A.* **115**, 7711–7716 (2018).
22. A. J. Kaufman, D. T. Johnston, J. Farquhar, A. L. Masterson, T. W. Lyons, S. Bates, A. D. Anbar, G. L. Arnold, J. Garvin, R. Buick, Late Archean biospheric oxygenation and atmospheric evolution. *Science* **317**, 1900–1903 (2007).
23. J. Garvin, R. Buick, A. D. Anbar, G. L. Arnold, A. J. Kaufman, Isotopic evidence for an aerobic nitrogen cycle in the latest Archean. *Science* **323**, 1045–1048 (2009).
24. L. V. Godfrey, P. G. Falkowski, The cycling and redox state of nitrogen in the Archaean ocean. *Nat. Geosci.* **2**, 725–729 (2009).
25. T. Bosak, A. H. Knoll, A. P. Petroff, The meaning of stromatolites. *Annu. Rev. Earth Planet. Sci.* **41**, 21–44 (2013).
26. B. Kendall, T. W. Dahl, A. D. Anbar, The stable isotope geochemistry of molybdenum. *Rev. Mineral. Geochem.* **82**, 683–732 (2017).

27. C. A. Miller, B. Peucker-Ehrenbrink, B. D. Walker, F. Marcantonio, Re-assessing the surface cycling of molybdenum and rhenium. *Geochim. Cosmochim. Acta* **75**, 7146–7179 (2011).
28. A. T. Greaney, R. L. Rudnick, R. M. Gaschnig, J. B. Whalen, B. Luais, J. D. Clemens, Geochemistry of molybdenum in the continental crust. *Geochim. Cosmochim. Acta* **238**, 36–54 (2018).
29. B. E. Erickson, G. R. Helz, Molybdenum(VI) speciation in sulfidic waters: Stability and lability of thiomolybdates. *Geochim. Cosmochim. Acta* **64**, 1149–1158 (2000).
30. B. C. Bostick, S. Fendorf, G. R. Helz, Differential adsorption of molybdate and tetrathiomolybdate on pyrite (FeS2). *Environ. Sci. Technol.* **37**, 285–291 (2003).
31. G. R. Helz, E. Bura-Nakić, N. Mikac, I. Ciglenečki, New model for molybdenum behavior in euxinic waters. *Chem. Geol.* **284**, 323–332 (2011).
32. C. Scott, T. W. Lyons, A. Bekker, Y. A. Shen, S. W. Poulton, X. L. Chu, A. D. Anbar, Tracing the stepwise oxygenation of the Proterozoic ocean. *Nature* **452**, 456–459 (2008).
33. C. Scott, T. W. Lyons, Contrasting molybdenum cycling and isotopic properties in euxinic versus non-euxinic sediments and sedimentary rocks: Refining the paleoproxies. *Chem. Geol.* **324–325**, 19–27 (2012).
34. C. Siebert, J. D. Kramers, T. Meisel, P. Morel, T. F. Nägler, PGE, Re-Os, and Mo isotope systematics in Archean and early Proterozoic sedimentary systems as proxies for redox conditions of the early Earth. *Geochim. Cosmochim. Acta* **69**, 1787–1801 (2005).
35. M. Wille, J. D. Kramers, T. F. Nägler, N. J. Beukes, S. Schröder, T. Meisel, J. P. Lacassie, A. R. Voegelin, Evidence for a gradual rise of oxygen between 2.6 and 2.5 Ga from Mo isotopes and Re-PGE signatures in shales. *Geochim. Cosmochim. Acta* **71**, 2417–2435 (2007).
36. Y. Duan, A. D. Anbar, G. L. Arnold, T. W. Lyons, G. W. Gordon, B. Kendall, Molybdenum isotope evidence for mild environmental oxygenation before the Great Oxidation Event. *Geochim. Cosmochim. Acta* **74**, 6655–6668 (2010).
37. A. R. Cabral, R. A. Creaser, T. Nägler, B. Lehmann, A. R. Voegelin, B. Belyatsky, J. Pašava, A. S. Gomes Jr., H. Galbiatti, M. E. Böttcher, P. Escher, Trace-element and multi-isotope geochemistry of Late-Archean black shales in the Carajás iron-ore district, Brazil. *Chem. Geol.* **362**, 91–104 (2013).
38. C. M. Ostrander, S. G. Nielsen, J. D. Owens, B. Kendall, G. W. Gordon, S. J. Romaniello, A. D. Anbar, Fully oxygenated water columns over continental shelves before the Great Oxidation Event. *Nat. Geosci.* **12**, 186–191 (2019).

39. S. L. Olson, L. R. Kump, J. F. Kasting, Quantifying the areal extent and dissolved oxygen concentrations of Archean oxygen oases. *Chem. Geol.* **362**, 35–43 (2013).
40. P. Liu, C. E. Harman, J. F. Kasting, Y. Hu, J. Wang, Can organic haze and O2 plumes explain patterns of sulfur mass-independent fractionation during the Archean? *Earth Planet. Sci. Lett.* **526**, 115767 (2019).
41. S. J. Daines, B. J. Mills, T. M. Lenton, Atmospheric oxygen regulation at low Proterozoic levels by incomplete oxidative weathering of sedimentary organic carbon. *Nat. Commun.* **8**, 14379 (2017).
42. A. C. Johnson, S. J. Romaniello, C. T. Reinhard, D. D. Gregory, E. Garcia-Robledo, N. P. Revsbech, D. E. Canfield, T. W. Lyons, A. D. Anbar, Experimental determination of pyrite and molybdenite oxidation kinetics at nanomolar oxygen concentrations. *Geochim. Cosmochim. Acta* **249**, 160–172 (2019).
43. M. Gleisner, R. B. Herbert Jr., P. C. F. Kockum, Pyrite oxidation by *Acidithiobacillus ferrooxidans* at various concentrations of dissolved oxygen. *Chem. Geol.* **225**, 16–29 (2006).
44. E. E. Stüeken, D. C. Catling, R. Buick, Contributions to late Archaean sulphur cycling by life on land. *Nat. Geosci.* **5**, 722–725 (2012).
45. H. D. Holland, Model for the evolution of the Earth's atmosphere, in *Petrologic Studies: A Volume to Honor A. F. Buddington*, A. E. J. Engel, H. L. James, B. F. Leonard, Eds. (Geological Society America, 1962), pp. 447–477.
46. B. Rasmussen, R. Buick, Redox state of the Archean atmosphere: Evidence from detrital heavy minerals in ca. 3250–2750 Ma sandstones from the Pilbara Craton, Australia. *Geology* **27**, 115–118 (1999).
47. X. Gu, P. J. Heaney, F. D. A. Aarão Reis, S. L. Brantley, Deep abiotic weathering of pyrite. *Science* **370**, eabb8092 (2020).
48. R. M. Gaschnig, R. L. Rudnick, W. F. McDonough, A. J. Kaufman, Z. Hu, S. Gao, Onset of oxidative weathering of continents recorded in the geochemistry of ancient glacial diamictites. *Earth Planet. Sci. Lett.* **408**, 87–99 (2014).
49. A. T. Greaney, R. L. Rudnick, S. J. Romaniello, A. C. Johnson, R. M. Gaschnig, A. D. Anbar, Molybdenum isotope fractionation in glacial diamictites tracks the onset of oxidative weathering of the continental crust. *Earth Planet. Sci. Lett.* **534**, 116083 (2020).
50. S. Li, W. D. Junkin, R. M. Gaschnig, R. D. Ash, P. M. Piccoli, P. A. Candela, R. L. Rudnick, Molybdenum contents of sulfides in ancient glacial diamictites: Implications for molybdenum

delivery to the oceans prior to the Great Oxidation Event. *Geochim. Cosmochim. Acta* **278**, 30–50 (2020).

51. J. Hao, D. A. Sverjensky, R. M. Hazen, Redox states of Archean surficial environments: The importance of H2,g instead of O2,g for weathering reactions. *Chem. Geol.* **521**, 49–58 (2019).
52. B. S. Gregory, M. W. Claire, S. Rugheimer, Photochemical modelling of atmospheric oxygen levels confirms two stable states. *Earth Planet. Sci. Lett.* **561**, 116818 (2021).
53. S. V. Lalonde, K. O. Konhauser, Benthic perspective on Earth's oldest evidence for oxygenic photosynthesis. *Proc. Natl. Acad. Sci. U.S.A.* **112**, 995–1000 (2015).
54. N. J. Planavsky, S. A. Crowe, M. Fakhraee, B. Beaty, C. T. Reinhard, B. J. Mills, C. Holstege, K. O. Konhauser, Evolution of the structure and impact of Earth's biosphere. *Nat. Rev. Earth Environ.* **2**, 123–139 (2021).
55. H. D. Holland, Volcanic gases, black smokers, and the Great Oxidation Event. *Geochim. Cosmochim. Acta*, **66**, 3811–3826 (2002).
56. J. Hao, A. H. Knoll, F. Huang, R. M. Hazen, I. Daniel, Cycling phosphorus on the Archean Earth: Part I. Continental weathering and riverine transport of phosphorus. *Geochim. Cosmochim. Acta* **273**, 70–84 (2020).
57. A. L. Zerkle, C. H. House, R. P. Cox, D. E. Canfield, Metal limitation of cyanobacterial N_2 fixation and implications for the Precambrian nitrogen cycle. *Geobiology* **4**, 285–297 (2006).
58. E. E. Stüeken, R. Buick, B. M. Guy, M. C. Koehler, Isotopic evidence for biological nitrogen fixation by molybdenum-nitrogenase from 3.2 Gyr. *Nature* **520**, 666–669 (2015).
59. A. D. Anbar, A. H. Knoll, Proterozoic ocean chemistry and evolution: A bioinorganic bridge? *Science* **297**, 1137–1142 (2002).
60. E. E. Stüeken, M. A. Kipp, M. C. Koehler, R. Buick, The evolution of Earth's biogeochemical nitrogen cycle. *Earth Sci. Rev.* **160**, 220–239 (2016).
61. C. T. Reinhard, N. J. Planavsky, L. J. Robbins, C. A. Partin, B. C. Gill, S. V. Lalonde, A. Bekker, K. O. Konhauser, T. W. Lyons, Proterozoic ocean redox and biogeochemical stasis. *Proc. Natl. Acad. Sci. U.S.A.* **110**, 5357–5362 (2013).
62. T. Goldberg, C. Archer, D. Vance, S. W. Poulton, Mo isotope fractionation during adsorption to Fe (oxyhydr)oxides. *Geochim. Cosmochim. Acta* **73**, 6502–6516 (2009).

63. C. M. Ostrander, B. Kendall, S. L. Olson, T. W. Lyons, G. W. Gordon, S. J. Romaniello, W. Zheng, C. T. Reinhard, M. Roy, A. D. Anbar, An expanded shale δ^{98}Mo record permits recurrent shallow marine oxygenation during the Neoarchean. *Chem. Geol.* **532**, 119391 (2020).
64. B. Kendall, G. W. Gordon, S. W. Poulton, A. D. Anbar, Molybdenum isotope constraints on the extent of late Paleoproterozoic ocean euxinia. *Earth Planet. Sci. Lett.* **307**, 450–460 (2011).
65. S. Metz, J. H. Trefry, Chemical and mineralogical influences on concentrations of trace metals in hydrothermal fluids. *Geochim. Cosmochim. Acta* **64**, 2267–2279 (2000).
66. C. G. Wheat, M. J. Mottl, M. Rudnicki, Trace element and REE composition of a low-temperature ridge-flank hydrothermal spring. *Geochim. Cosmochim. Acta* **66**, 3693–3705 (2002).
67. J. McManus, T. Nägler, C. Siebert, C. G. Wheat, D. E. Hammond, Oceanic molybdenum isotope fractionation: Diagenesis and hydrothermal ridge-flank alteration. *Geochem. Geophys. Geosyst.* **3**, 1078 (2002).
68. R. L. Rudnick, S. Gao, Composition of the continental crust, in *Treatise on Geochemistry*, R. L. Rudnick, Ed. (Elsevier, 2003), vol. 3, pp. 1–64.
69. N. D. Greber, I. S. Puchtel, T. F. Nägler, K. Mezger, Komatiites constrain molybdenum isotope composition of the Earth’s mantle. *Earth Planet. Sci. Lett.* **421**, 129–138 (2015).
70. M. Willbold, T. Elliott, Molybdenum isotope variations in magmatic rocks. *Chem. Geol.* **449**, 253–268 (2017).
71. C. Alibert, M. T. McCulloch, Rare earth element and neodymium isotopic compositions of the banded iron-formations and associated shales from Hamersley, western Australia. *Geochim. Cosmochim. Acta* **57**, 187–204 (1993).
72. D. Asael, F. L. H. Tissot, C. T. Reinhard, O. Rouxel, N. Dauphas, T. W. Lyons, E. Ponzevera, C. Liorzou, S. Chéron, Coupled molybdenum, iron and uranium stable isotopes as oceanic paleoredox proxies during the Paleoproterozoic Shunga Event. *Chem. Geol.* **362**, 193–210 (2013).
73. D. Asael, O. Rouxel, S. W. Poulton, T. W. Lyons, A. Bekker, Molybdenum record from black shales indicates oscillating atmospheric oxygen levels in the early Paleoproterozoic. *Am. J. Sci.* **318**, 275–299 (2018).
74. S. Eroglu, R. Schoenberg, M. Wille, N. Beukes, H. Taubald, Geochemical stratigraphy, sedimentology, and Mo isotope systematics of the ca. 2.58–2.50 Ga-old Transvaal Supergroup carbonate platform, South Africa. *Precambrian Res.* **266**, 27–46 (2015).

75. B. Kendall, C. T. Reinhard, T. W. Lyons, A. J. Kaufman, S. W. Poulton, A. D. Anbar, Pervasive oxygenation along late Archaean ocean margins. *Nat. Geosci.* **3**, 647–652 (2010).
76. F. Kurzweil, M. Wille, R. Schoenberg, H. Taubald, M. J. Van Kranendonk, Continuously increasing δ^{98}Mo values in Neoarchean black shales and iron formations from the Hamersley Basin. *Geochim. Cosmochim. Acta* **164**, 523–542 (2015).
77. C. Manikyamba, R. Kerrich, Geochemistry of black shales from the Neoarchaean Sandur Superterrane, India: First cycle volcanogenic sedimentary rocks in an intraoceanic arc–trench complex. *Geochim. Cosmochim. Acta* **70**, 4663–4679 (2006).
78. S. M. McLennan, S. R. Taylor, K. A. Eriksson, Geochemistry of Archean shales from the Pilbara Supergroup, Western Australia. *Geochim. Cosmochim. Acta* **47**, 1211–1222 (1983).
79. S. M. McLennan, S. R. Taylor, T. A. Kröner, Geochemical evolution of Archean shales from South Africa. I. The Swaziland and Pongola Supergroups. *Precambrian Res.* **22**, 91–124 (1983b).
80. S. M. McLennan, S. R. Taylor, V. R. McGregor, Geochemistry of Archean metasedimentary rocks from West Greenland. *Geochim. Cosmochim. Acta* **48**, 1–13 (1984).
81. F. O. Ossa, A. Hofmann, M. Wille, J. E. Spangenberg, A. Bekker, S. W. Poulton, B. Eickmann, R. Schoenberg, Aerobic iron and manganese cycling in a redox-stratified Mesoarchean epicontinental sea. *Earth Planet. Sci. Lett.* **500**, 28–40 (2018).
82. B. F. F. Reczko, "The geochemistry of the sedimentary rocks of the Pretoria Group, Transvaal sequence," thesis, University of Pretoria (1996).
83. S. K. Sahoo, N. J. Planavsky, B. Kendall, X. Wang, X. Shi, C. Scott, A. D. Anbar, T. W. Lyons, G. Jiang, Ocean oxygenation in the wake of the Marinoan glaciation. *Nature* **489**, 546–549 (2012).
84. K. E. Yamaguchi, Geochemistry of Archean–Paleoproterozoic black shales: The early evolution of the atmosphere, oceans, and biosphere, thesis, Pennsylvania State University, State College (2002).
85. L. E. Wasylenki, B. A. Rolfe, C. L. Weeks, T. G. Spiro, A. D. Anbar, Experimental investigation of the effects of temperature and ionic strength on Mo isotope fractionation during adsorption to manganese oxides. *Geochim. Cosmochim. Acta* **72**, 5997–6005 (2008).
86. M. Wille, O. Nebel, M. J. Van Kranendonk, R. Schoenberg, I. C. Kleinhanns, M. J. Ellwood, Mo–Cr isotope evidence for a reducing Archean atmosphere in 3.46–2.76 Ga black shales from the Pilbara, Western Australia. *Chem. Geol.* **340**, 68–76 (2013).
